# Psychological differences in food addiction and binge eating in a general Polish population

**DOI:** 10.1038/s41598-025-87057-w

**Published:** 2025-01-31

**Authors:** Jagoda Różycka, Ari Nowacki, Marta Łukowska, Maryla Sokołowska, Joanna Zielińska, Roksana Duszkiewicz, Monika M. Stojek

**Affiliations:** 1 Institute of Psychology, University of Silesia, ul. Grażyńskiego 53, Katowice, 40-126 Poland; 2 SWPS University of Social Sciences and Humanities, Katowice, Poland; 3 Faculty of Medicine, Academy of Silesia, Katowice, Poland; 4 Department of Psychiatry and Behavioral Sciences, Emory University School of Medicine, Atlanta, GA USA

**Keywords:** Food addiction, Binge eating, Eating disorders, Dysregulated eating, Psychological functioning, Questionnaire, Emotional symptoms, Obesity, Poland, Psychology, Health care

## Abstract

The aim of the study was to identify the prevalence of food addiction (FA) and binge eating (BE) in a general Polish population, to explore the differences between these constructs, and to examine psychological differences among FA, BE, and FA + BE groups. 2123 participants completed the questionnaires: Yale Food Addiction Scale 2.0, Binge Eating Scale, The PTSD Checklist for DSM-5, Life Events Checklist, Adverse Childhood Experiences, Short UPPS-P scale (impulsive traits), Dutch Eating Behavior Questionnaire and Depression, Anxiety and Stress Scale − 21 items. 492 participants reported clinically significant symptoms of FA, BE, or both. Spearman’s correlations, Mann-Whitney U tests, and Kruskal-Wallis tests were performed to examine the relationships between variables. The present study showed that FA and BE symptoms are common in the adult sample. The FA, BE and FA + BE groups had higher scores than the control group for all variables included in the study. The FA + BE group presented a significantly higher level of behavioral and emotional symptoms, followed by the FA and the BE group with less severity of symptoms. This study supports the hypothesis of food addiction as a similar construct to BE. FA is a broader concept than BE, but individuals experiencing both indicated the greatest functional difficulties.

**Supplementary Information:**

The online version contains supplementary material available at 10.1038/s41598-025-87057-w.

## Introduction

Excessive food consumption and its results, like eating disorders (EDs) or obesity^1^ are a worldwide problem. In addition to the clinical population, the phenomenon also occurs among people with disordered eating behaviors (DEBs) - or subclinical problematic eating behaviors – who might not see it as a significant difficulty^2,3^. A growing body of research emphasizes the importance of exploring DEBs, including binge eating (BE). In previous studies, clinical EDs were not specifically excluded, so current knowledge of subclinical patterns of DEBs might be limited.

BE is defined as the occurrence of sudden episodes of excessive food consumption in a discrete period of time with a sense of lack of control over eating^4^. The episodes may be preceded by emotional distress and could be a strategy to avoid disturbing thoughts and emotions^5,6^. While loss of control characterizes BE, this phenomenon differs from the concept of loss of control in addictions^7^. An uncontrolled binge is explained by a combination of nutritional deprivation and psychological mechanisms, such as violating dietary restraints^8^. BE might evolve into a binge eating disorder (BED), but many people declare subclinical symptoms that cause significant distress^2^.

Some individuals describe binge eating as a form of “food addiction” (FA)^9^. FA is defined as increased compulsive food intake, which in turn may lead to the loss of its hedonic value - a reduction in the enjoyment or pleasure derived from eating^4,10^. Similar to other types of addiction, individuals with FA often consume larger amounts of food than intended and experience cravings, urges, or a strong desire to eat. They often report spending large amounts of time obtaining food, eating, or recovering from eating. Additionally, they mention a persistent desire for food and unsuccessful attempts to cut down on eating. They may report the necessity to eat more to reduce negative emotions or to increase pleasure, as well as withdrawal symptoms such as experiencing negative affect or physical symptoms when they stop eating. As with any addiction, FA causes clinically significant impairment and distress^11,12^.

Overeating may vary in the severity of symptoms and the degree of compulsiveness. Davis^4^ proposed a continuous spectrum of eating difficulties. At one end of the continuum, there is passive overeating with no clinical consequences, then binging without clinical impairment (rare and without somatic consequences), intermittent binge eating (often with all BED symptoms), and at the other end - those who meet the proposed criteria for FA based on the Yale Food Addiction Scale (YFAS)^13^, with higher severity of BED symptoms. Davis^4^ proposed that persons with FA also express significantly more severe and compulsive symptomatology and psychopathological characteristics. In particular, they are highly impulsive, manifest significant mood disturbances, and display emotionally driven bouts of overeating in comparison to the BE group. It may be seen as an FA phenomenon – with nearly all symptoms of FA present in BE and BED patients but in higher severity. To check if FA might be better understood as severe BE and if it differs from other ED patterns^4^, further research is needed on a larger sample.

Individuals prone to BE may consume more food than planned and experience intense cravings for food^9^. BE may result in being overweight, but it is not a rule - individuals may not see BE as a problem due to maintaining healthy weight. A meta-analysis conducted by Bao et al.^14^ found a small yet significant positive association between FA and BMI, as well as significant associations between FA, objective BE, and ED psychopathology. Despite studies showing the overlap of FA and BE in people with obesity^15^, there is little research on FA and BE combined symptoms. The prevalence of FA ranges from 42 to 57% in populations with BE and BED, further demonstrating the differences between the two constructs^16^. So, if we conclude that in some patients with BE, it might be understood as FA, the intervention type may change. The presence of an addictive component in some subjects affected by ED might influence their treatments and outcomes if not correctly addressed.

Studies suggest a link between FA and increased BMI^15^ as well as emotional dysregulation, low self-esteem, and depression in the BE + FA group^14^. Factors like early-life stress, trauma, and negative urgency may influence BE and FA symptoms, affecting eating behaviors and psychological functioning. Furthermore, constructs such as emotional or restrictive eating, stress reactions, emotional functioning, and negative urgency may be crucial for the relationship between the BE symptoms continuum identified by Davis^4^ as they might differentiate the FA from the BE spectrum. According to a meta-analysis of cross-sectional studies, there are generally positive, moderate associations between FA, BE symptoms, depression, and anxiety^16–18^.

Factors like early-life stress^19,20^, trauma^21^, and negative urgency^22–24^ may influence BE and FA symptoms, affecting eating behaviors and psychological functioning. The early-life stressors (or traumatic events), and various dietary differences that arise from these stressors are influential in the susceptibility to compulsive intake of palatable foods^19^. Moreover, PTSD appears to be related to ED symptoms, FA, emotion suppression, and cognitive distortions^25,26^.

It is believed that individuals with BE, FA, and the co-occurrence of FA and BE may exhibit variations in psychological functioning, leading to different responses to interventions that are not tailored to their specific DEBs. The ongoing debate on whether BE can be considered FA or if FA is a subtype of BE remains an area of active research. The distinct differences between BE and FA are not as well understood together with their psychological characteristics (including posttraumatic events’ consequences), underscoring the importance of characterizing these constructs. What is more, little is known about the psychological characteristics of the mixed DEBs group and whether they differ from BE or FA alone.

The main aim of the study was to explore the differences in psychological characteristics between FA, BE and mixed eating pathology group (both FA + BE) in a Polish adult population. Specifically, groups were compared on BMI, eating-related variables (including restrained eating and emotional eating), internalizing symptoms (specifically depression and anxiety severity), stress-related variables (stress severity, PTSD symptoms, adverse childhood events, and the number of traumatic events), and negative urgency. We also compared the groups within the ED group - i.e., FA, BE, and co-occurring FA + BE – on these psychological variables to determine whether individuals who belong to each of these groups differ in the severity of other psychological symptoms. The study design and sample selection were aimed at contributing to the BE/FA research, especially in gathering the Polish representative general sample, including persons with co-occurring FA + BE symptoms, and including representative groups of both women and men, which has often been lacking in previous studies.

## Methods

### Participants and procedure

This study is part of a larger project concerning posttraumatic stress disorder (PTSD), food addiction, and health. We estimated the sample size to be about 2000 people. Due to the fact that it was a screening group for the study of PTSD and FA symptoms, a large sample size was necessary to recruit symptomatic individuals given prevalence statistics (6–12% of EU citizens have PTSD^27,28^, 8–10% of the general population have FA in the United States and Germany^29,30^). Participants between ages 18 and 55, living in Poland, were invited to complete an online survey on FA and BE, which took approximately 30 min to complete. The age criteria were set due to the assumptions of the whole project about cardiometabolic health (55 as the upper limit, which minimizes the risk of cardiac or metabolic diseases). Exclusion criteria included being pregnant/currently lactating and being unable to speak and read the Polish language. The recruitment process used various strategies: sending invitations through the university’s newsletter, the project’s social media and website, and paid advertisement in a local newspaper. Additionally, a snowball method was applied, and an external pollster company (Nationwide Research Panel Ariadna; https://panelariadna.pl/*)* was employed. Participants could fill in the questionnaire either on an online platform LimeSurvey or through the pollster’s company platform. A monetary compensation using a lottery system was offered to participants who completed the survey using LimeSurvey. The participants who used the pollster’s company platform collected points for completing the survey that could be exchanged for prizes. Informed consent was obtained from all subjects before taking the survey. This study was approved by The University of Silesia Ethics Committee (document of approval number: KEUS 245/04.2022, Katowice, Poland).

### Measures

#### Sociodemographic variables

Participants provided information on their gender, age, and highest level of education achieved. Additionally, participants reported their employment status, marital status, monthly income, and place of residence. They also indicated the presence of somatic diseases and psychiatric diagnoses, specifying which diagnoses from the presented list, if any, were present. Similarly, participants answered questions about medication by responding ‘YES’ or ‘NO’ on a list of different types of drugs. Finally, participants answered a question about smoking, with response options: ‘Not smoking,’ ‘Smoking,’ and ‘Not smoking for a month’.

#### Outcome variables

The Yale Food Addiction Scale (YFAS 2.0^13,31^) is a 35-item self-report measure that allows to assess eleven symptoms of food addiction experienced over the last 12 months. The YFAS 2.0 asks participants to think of specific foods, such as highly processed foods; however, participants in this study were asked to consider all foods. Participants respond using a scale from 0 “Never” to 7 “Every day”. The total YFAS score was computed as a symptom count ranging from 0 to 11. When two or more symptoms are endorsed that cause a clinically significant impairment a “diagnosis” of FA can be made. In the current study, the internal consistency was α = 0.95.

Binge Eating Scale (BES^32^, Polish translation by Różycka, unpublished) is a 16-item questionnaire used to assess binge eating behavior. Each question requires choosing from three to four possible responses, reflecting a range of severity of binge eating behaviors. Participants were shown questions related to BE after they answered “Yes” to the question concerning experiencing subjective binge eating episodes. Each of the responses is weighted, with the total score being a sum ranging from 0 to 46. Participants with scores above 17 are classified as having a problem with BE. The questionnaire was translated two-way by a fluent native speaker, and the consistency in the current study was = 0.86.

Participants who scored above the cut-off point for both the YFAS and BES scales were sorted into the double diagnosis group.

#### Emotional symptoms

Depression, Anxiety and Stress Scale – 21 items (DASS-21^33,34^) is a 21-item questionnaire that assesses depression, anxiety, and stress symptoms over the prior week. Participants answer using a 4-point Likert scale ranging from 0 “Did not apply to me at all” to 3 “Applied to me very much, or most of the time”. The scores for each subscale are obtained by summing corresponding items, with results ranging from 0 to 21. In the current study, the Cronbach’s alpha for this tool was α = 0.92 for Stress, α = 0.87 for Anxiety, and α = 0.93 for Depression.

#### Eating-related variables

Restrained Eating and Emotional Eating are subscales from the Dutch Eating Behaviour Questionnaire (DEBQ^35,36^) that assess participants’ eating behaviors. Emotional Eating consists of 13 items concerning eating as a response to emotional states, while Restrained Eating comprises 10 items concerned with eating smaller amounts of food than wanted. Participants rate their experiences on a 5-point Likert scale ranging from 1 “Never” to 5 “Very often”. For each subscale, a score is calculated as a mean of the corresponding items. In the current study, the Cronbach alpha for Restrained Eating was α = 0.93 and for Emotional Eating α = 0.97.

Body mass index (BMI) is a person’s weight in kilograms divided by the square of height in meters. In the current study, we relied on self-reported measures of weight and height of participants.

#### Stress-related variables

Adverse Childhood Experiences Scale (ACE^37^) consists of 10 items concerning exposure to trauma during childhood/adolescence. Respondents provide 1 “Yes” or 0 “No” responses, which are summed to form the “ACE” Score ranging from 0 to 10. A professional translator translated this questionnaire into Polish.

The PTSD Checklist for DSM-5 (PCL-5^38,39^) is a 20-item self-report measure of the DSM-5 symptoms of PTSD. The respondents rate the items on a 5-point Likert scale ranging from 0 “not at all” to 4 “Extremely”. The total score is calculated as a sum of all the items, ranging from 0 to 80. In the current study, the Cronbach alpha for PCL-5 was α = 0.96.

The Life Events Checklist (LEC-5^40,41^) is used to assess individuals’ exposure to 16 traumatic events according to DSM-IV criteria. Respondents rate their experience on a multiple-choice, nominal scale (1 “Happened to me”, 2 “Witnessed it”, 3 “Learned about it”, 4 “Not sure”, 5 “Doesn’t apply”). The LEC score is calculated as the number of events experienced and witnessed by participants^42^, with the score ranging from 0 to 32.

#### Impulsivity

Negative urgency is a subscale of The Short Impulsive Behaviour Scale (SUPPS-P^43^) that assesses acting impulsively under the influence of negative affect. The subscale consists of 4 items, which participants rate on a 5-point Likert scale ranging from 0 “I totally agree” to 4 “I totally disagree. The final score for the subscale ranges from 0 to 16, with higher scores indicating higher intensity of the negative urgency. In the current study, the internal consistency was α = .82.

### Data analysis

Data were coded and analyzed using IBM SPSS software. A preliminary analysis of frequency histograms and Shappiro-Wilk test results indicated a violation of a normal distribution for all of the study variables^44^ (see: Table A in Appendix). First, we analyzed the relationship between the variables using Spearman’s correlation as a method suitable for datasets with a high number of extreme results^45^. Spearman’s rho measures monotonic relationships that may not be linear in nature^46^. Then, based on an analysis of the variables’ Q-Q plots that indicated a deviation from normality^44,47^, we used Mann-Whitney’s test to explore differences between the control group and the eating disorder group (comprising individuals with FA, BE, or FA + BE) and Kruskal-Wallis test to evaluate the differences between the FA, BE, and FA + BE groups. A posthoc tests used to check differences between particular groups. Additionally, we applied the Bonferroni correction to adjust for multiple comparisons.

## Results

### Sample characteristics

The final sample comprised 2123 participants who completed the study, among whom 492 individuals met the criteria for either FA, BE, or both (based on YFAS or BES score; see: Measures). There were *n* = 182 (8.57%) persons with FA, *n* = 148 (6.97%) with BE, and *n* = 162 (7.63%) with both disorders. A comparison group without a disordered eating pattern consisted of *n* = 1631 (76.83%) participants. The final sample characteristics are shown in Table 1. The majority of the participants were female (58.9%), with a mean age of 22.06 (SD = 16.60). Most of the sample had higher education and a full-time job.

**Table 1:** Sociodemographic characteristics of the sample.

Sociodemographic variable	Study sample (*n* = 2123) (%)
Gender	
Male	41.00
Female	58.32
Other	0.68
Age	
18–24 years of age	21.15
25–34 years of age	24.31
35–44 years of age	21.81
45–55 years of age	32.74
Education	
Primary	1.7
Vocational	7.2
Secondary	37.15
Tertiary	53.69
Employment	
Unemployed	9.69
Student	19.48
Employed full-time	56.22
Employed part-time	7.75
Employed contract	6.86
Marital status	
Single	43.92
Married	38.70
Divorced	5.55
In cohabitation	11.32
In separation	0.54
Income	
Under 2000 PLN	8.30
Between 2001 and 3500 PLN	14.43
Between 3501 and 6000 PLN	27.19
Between 6001 and 8000 PLN	19.43
Between 8001 and 10 000 PLN	14.65
Above 10 000 PLN	16.00
Place of residence	
Countryside	19.43
Small town (less than 50.000 citizens)	18.21
Middle-sized town (between 50 000 and 150 000 citizens)	21.60
City (between 150.000 and 500 000 citizens)	32.82
Big city (above 500 000 citizens)	7.93

The health status of participants is presented in Table 2. The mean of the BMI 25.97 (SD = 5.70, Min = 14.87, Max = 75.69). The majority of the sample reported having at least one psychiatric diagnosis and not participating in psychotherapy. Among the most prevalent diagnoses were mood disorders, anxiety disorders, hypertension, and obesity.

**Table 2:** Health status of participants.

Health variable	Study sample (*n* = 2123) (%)
Smoking	
No	61.33
Yes	27.41
Not smoking for a month	11.26
Somatic diseases	
Hypertension	15.87
Diabetes type II	4.19
Insulin resistance	5.51
Digestive system diseases	6.31
Anemia	3.67
Obesity	12.01
Hypothyroidism	1.74
Hyperthyroidism	9.14
Autoimmune diseases	2.5
No diagnosis	52.38
Current psychotherapy attendance	
Yes	10.17
Psychiatric diagnoses	
Mood disorders	13.00
Anxiety disorders	13.37
Eating disorders	4.38
Stress-related disorders	13.09
Psychotic	2.17
No diagnosis	72.96
Medications	
Drugs for mood	13.61
Supplements	34.86
Hypertension	13.05
Diabetes	5.18
No medications	39.42

### Correlation between study variables

We analyzed the relationship between the study variables using Spearman’s correlation. The results are presented in Table A (see the Supplementary Materials). YFAS score has shown a positive moderate relationship with BES score (*rho* = 0.52, *p* < .001 ), emotional eating (*rho* = 0.63, *p* < .001), depression (*rho* = 0.44, *p* < .001), anxiety (*rho* = 0.49, *p* < .001), stress (*rho* = 0.46, *p* < .34), and PCL score (*rho* = 0.49, *p* < .001 ). On the other hand, BES scores had a moderate positive relationship only with emotional eating (*rho* = 0.49, *p* < .001) and weak positive relationships with all of the remaining variables. Of note, BMI had a weak positive relationship with both YFAS (*rho* = 0.14, *p* < .001) and BES scores (*rho* = 0.15, *p* < .001).

### Group comparison – ED and the control group

The results of the U Mann-Whitney test for the control group and ED group are shown in Table 3. There were differences in the YFAS score (U = 67092.50, *p* < .001), BES score (U = 114808.5, *p* < .001), BMI (U = 331720.50, *p* < .001), restrained eating (U = 266641.00, *p* < .001), emotional eating (U = 124120.50, *p* < .001), depression (U = 207610.50, *p* < .001), anxiety (U = 201362.50, *p* < .001), stress (U = 191156.00, *p* < .001), negative urgency (U = 281281.00, *p* < .001), ACE (U = 275653.00, *p* < .001), PCL (U = 281180.50, *p* < .001), and LEC scores (U = 185889.50, *p* < .001). For all those scales, participants with at least one eating disorder achieved significantly higher scores.

**Table 3:** Descriptive statistics of the study variables.

	Control group	ED Group	Min	Max	U
	Me	Me			
YFAS score	0.00	7.00	0.00	11.00	67092.50.00***
BES score	0.00	7.00	0.00	46.00	114808.50***
BMI	24.69	26.06	14.87	62.24	331720.50***
Restrained Eating (DEBQ)	2.55	3.00	1.00	5.00	266641.00***
Emotional Eating (DEBQ)	1.92	3.23	1.00	5.00	124120.50***
Depression (DASS-21)	5.00	11.00	0.00	21.00	207610.50***
Anxiety (DASS-21)	3.00	9.00	0.00	21.00	201362.50***
Stress (DASS-21)	7.00	12.00	0.00	21.00	191156.00***
ACE	0.00	1.00	0.00	10.00	275653.00***
PCL-5	23.00	45.00	0.00	80.00	185889.50***
LEC	4.00	8.00	0.00	51.00	281180.50***
Negative urgency	10.00	11.00	4.00	16.00	281281.00***

*YFAS score* Yale Food Addiction Scale number of symptoms, *BES* Binge Eating Scale, *BMI* Body Mass Index, *ACE* Adverse Childhood Experiences Scale, *PCL-5* The PTSD Symptom Checklist for DSM-5, *LEC* The Life Events Checklist.

***p < .001..

### Group comparison – FA, BE, and double diagnosis

The results of the Kruskall-Wallis test are presented in Table 4. People with a double diagnosis had significantly higher levels than the BE group on the YFAS scores (*p* < .001), BES scores (*p* < .001), negative urgency (*p* = .029), emotional eating (*p* < .001), restrained eating (*p* = .002), depression (*p* < .001), anxiety (*p* = .028), stress (*p* < .001), ACE (*p* = .039), and PCL (*p* < .001). Additionally, the double diagnosis group had higher scores than the FA group on YFAS score (*p* = .007), BES score (*p* < .001), negative urgency (*p* = .010), emotional eating (*p* < .001), ACE (*p* < .001), and LEC (*p* = .028). The FA group, compared to the BE group, had higher levels of the YFAS score (*p* < .001), anxiety (*p* = .0028), stress (*p* < .001), and PCL (*p* < .001). Participants in the BE group reported higher scores on the BES (*p* < .001) and had a higher BMI (*p* = .049) than the FA group. None other comparisons were significant after the Bonferroni adjustment.

**Table 4:** The results of the Kruskal – Wallis test for FA, BE, and FA + BE groups.

	FA (1) *n* = 182	BE (2) *n* = 148	FA + BE (3) *n* = 162	H	df	*p*	Sig. Comparisons
	Me	Me	Me				
YFAS score	7.00	5.00	9.00	66.86	2	< 0.001	3 > 1 > 2
BES score	0.00	22.00	25.00	358.52	2	< 0.001	3 > 2 > 1
BMI	25.86	27.24	26.07	6.49	2	0.049	2 > 1
Restrained eating	3.00	2.91	3.09	11.68	2	0.003	3 > 2
Emotional eating	3.00	3.04	3.69	31.82	2	< 0.001	3 > 2; 3 > 1
Depression	11.00	10.00	12.50	14.73	2	< 0.001	3 > 2
Anxiety	9.50	8.00	9.00	8.09	2	0.017	1 > 2; 3 > 2
Stress	12.00	10.50	12.50	20.34	2	< 0.001	1 > 2; 3 > 2
ACE	1.00	2.00	2.00	28.21	2	< 0.001	2 > 1; 3 > 1; 3 > 2
PCL	46.00	40.00	48.00	32.07	2	< 0.001	1 > 2; 3 > 2
LEC	7.00	8.00	9.00	6.80	2	0.033	3 > 1
Negative urgency	11.00	11.00	12.00	10.83	2	0.004	3 > 1; 3 > 2;

*YFAS score* Yale Food Addiction Scale number of symptoms, *BES* Binge Eating Scale, *ACE* Adverse Childhood Experiences Scale, *PCL* The PTSD Checklist for DSM-5, *LEC* The Life Events Checklist, *Sig. Comparisons* significant comparisons.

***p < .001; **p < .01; *p < .05..

## Discussion

The primary goal of this study was to examine the psychological correlates of FA, BE and a combination of both (FA + BE) in the Polish adult population. We demonstrated that FA and BE symptoms are present at relatively high rates among Polish adults. As expected, compared to individuals without reported disordered eating behaviors (DEBs), individuals with these symptoms demonstrated greater severity of eating difficulties (i.e., emotional eating and dietary restraint), higher BMI, more emotional symptoms (depression, anxiety, stress), more PTSD symptoms severity in the clinical range (vs. a non-clinical range for control participants), as well as reported more lifetime traumatic events and adverse childhood events (as Brewerton^48^ and Tabone^21^ suggested). Within the DEB groups, we found that the FA + BE group presented the more pronounced pattern of behavioral and emotional symptoms, followed by the FA alone group, while the BE group had the fewest symptoms. Individuals with FA + BE, compared to either one of the other groups, reported significantly more FA and BE symptoms, greater emotional eating, and more adverse childhood events. They also reported higher levels of restrained eating, depression, anxiety, stress, and negative urgency, as well as more severe PTSD symptoms compared to the BE group alone. The differences between FA and BE were less pronounced when compared to the FA + BE group.

Participants with FA + BE were more likely to overeat for emotional reasons. It has been reported that negative affect is a key feature across cognitive-behavioral theories of binge eating^49^. One proposed mechanism is that binge eating serves as an avoidance from awareness^50^ in which the salience of adverse emotions is limited through cognitive narrowing that occurs while consuming food. There has been empirical evidence that negative mood plays a significant role in binge eating^51^ – as a consequence but in addition as a trigger for binging. The results are also in agreement with those of Stice et al.^58^- the dietary/depressed subgroup in their study exhibited mental dietary preoccupation, increased interpersonal sensitivity, negative urgency, alexithymia, and lower emotional regulation strategies. In the current study, compared to the BE participants, the FA group reported slightly more negative consequences due to their eating difficulties and had significantly elevated symptoms of anxiety and stress, which is in line with previous research by Parylak et al.^52^. According to Burrows et al.^17^, FA might be a result of coping with distress through eating. The FA group also reported heightened PTSD symptoms, suggesting potential factors contributing to the development and/or maintenance of compulsive food-seeking behaviors.

We observed that the FA + BE group had markedly more pronounced symptoms than FA or BE separately. As we previously noted, FA and BE share many common features^53^. The studies by Davis et al.^54^ and Gearhardt et al.^55^ found that more than half of obese adults diagnosed with FA (based on YFAS) also met the criteria for BE. We observed that people with FA presented slightly more impairment in psychological functioning than people with high scores on the BES scale without FA. FA might represent a medium stage on the BE psychopathology continuum but it is a very cautious suggestion worth further studies. These results might be in line with the eating disorders continuum defined by Davis^4^ and the reward-based process model connecting negative emotional states and food addiction^56^. We might hypothesize that the FA + BE group represents addiction to quantity and specific types of food.

Despite the high anxiety symptoms associated with binge eating overall^57^, research suggests that binge eating itself is associated with reduced anxiety^58^ as a binge eating episode is followed by lower levels of the experienced symptoms. In our research, we might speculate that while BE may be more connected to depressed mood instead of anxiety, the FA group - being slightly more anxious and stressed - implemented ineffective regulation strategies with eating becoming an urge and conditioned habit with irregular psychological rewards. This reasoning is in line with seeing FA through the lens of addiction disorders. Such a hypothesis needs more research implementing different methodologies due to the cross-sectional character of our research.

Participants with FA symptoms had higher PTSD symptoms than the BE group. There might be an overlap in mechanisms between EDs and PTSD, as concluded in Meule and Gearhardt’s review^59^. People with PTSD have a higher prevalence of all EDs^,^ especially those connected to binging^60^. Trottier and MacDonald^61^ conclude that the potential mechanisms of the trauma-ED relationship may lie in emotional dysregulation, anger, and impulsivity/compulsivity. In Brewerton’s review^42^, it was stated that experiencing ACEs may lead to self-destructive behaviors, including ED behaviors. Contrary to this, FA participants had fewer ACE’s and LEC symptoms, so it may be hypothesized that FA may not serve as a regulation strategy, more like compulsivity and reward-seeking behaviors.

Finally, we noted that the BE group had a higher BMI than the FA group. The correlation analyses (reported in the Supplementary Materials) indicated that the relationship between FA symptoms, BE severity, and BMI was very weak. Cernelic-Bizjak and Guine^62^ proposed that uncontrolled eating and BMI might be separate phenomena that affect one another only in certain cases. The results of this study demonstrated that adults with FA or FA + BE may not have obesity and still report symptoms of uncontrolled eating and their consequences. We must note that compensatory behaviors were not controlled and may influence the overall weight of the participants. However, other factors contributing to uncontrolled eating and BMI should also be considered. It is possible that potential BMI-influencing factors like the level of everyday spontaneous activity, type of work, dietary habits, medication use, and biological predispositions affected the relationship between disordered eating and BMI. In the group comparison, people with FA and BE in the ED group had a slightly higher average BMI than those in the control group. Due to the subclinical character of the research sample, they may represent a risk group for future weight gain and require prevention interventions.

Some limitations of the present study should be mentioned. Due to the cross-sectional study design, it was impossible to investigate causal relationships between variables. Also, the online version of the research might have had an impact on the participants’ responses, as external stimuli might have influenced their ability to focus on the questionnaire. Furthermore, as we only used self-reports, we did not have an objective measure of participants’ height and weight to generate measured BMI as well as access to the history of weight along with the dietary history. Moreover, not all participants filled out BES as it was an optional questionnaire for individuals who endorsed loss of control over eating. Also, it is not a method for BED diagnosis, which excludes the possibility of discussing the binge eating disorder symptoms at a confirmed clinical level. Furthermore, caloric intake and activity levels were not controlled. Those aspects are relevant in analyzing the binge behavior structure, as it is possible that some percentage of binges might be the result of excessive activity or low caloric intake during the day. Further studies should consider compensatory behaviors.

Despite the limitations, the strength of the study is the novelty of FA, BE, and FA + BE comparison in a large sample of adults without pronounced obesity and the observed differences between the persons with high levels of symptoms of FA, BE or both. Moreover, a wide set of psychological characteristics and gender variance were taken into account in the sample.

## Conclusions

The study provides further support for the usefulness of FA diagnosis when identifying the BE features in DEBs. Due to the fact that the FA + BE group was markedly more impaired and experienced more severe psychological symptoms, it might be recommended to check FA symptoms when assessing a history of binges. They may be often diagnosed together, but due to the behavior structure and slightly different symptoms, professionals may have to match the effective treatment. Treatment of BE could be ineffective if we do not take potential FA symptoms into account. Of note, disordered eating behaviors might not be related to higher BMI, but they might be a risk factor for developing obesity in the future. It constitutes another reason for the prevention of FA development among people with BE.

## Electronic supplementary material

Below is the link to the electronic supplementary material.

Supplementary Material 1

## Data Availability

For the purpose of Open Access, the author has applied a CC-BY public copyright license to any Author Accepted Manuscripts (AAM) version arising from this submission. The datasets generated during and/or analysed during the current study are available from the co-author upon request (for. M.M.Stojek). The dataset will be stored in the Open Science Framework repository and will be publicly available by January 31, 2025.
