# Supplementary material for: Psychological differences in food addiction and binge eating in a general Polish population
Source: Sci Rep. 2025 Jan 31;15:3919. doi: 10.1038/s41598-025-87057-w (PMC11785786; doi:10.1038/s41598-025-87057-w)
Supplement: Supplementary file 1 — Supplementary Material 1 [file 41598_2025_87057_MOESM1_ESM.docx]

**Supplementary materials**

Table A

Spearman’s correlations of the study’s variables

|  | 1. | 2. | 3. | 4. | 5. | 6. | 7. | 8. | 9. | 10. | 11. | S-W |
| --- | --- | --- | --- | --- | --- | --- | --- | --- | --- | --- | --- | --- |
| YFAS score |  |  |  |  |  |  |  |  |  |  |  | .94*** |
| BES score | .52*** |  |  |  |  |  |  |  |  |  |  | .89*** |
| BMI | .14*** | .15*** |  |  |  |  |  |  |  |  |  | .91*** |
| Restrained eating | .27*** | .24*** | .18*** |  |  |  |  |  |  |  |  | .99* |
| Emotional eating | .63*** | .49*** | .11*** | .39*** |  |  |  |  |  |  |  | .98*** |
| Depression | .44*** | .31*** | -.04 | .16*** | .42*** |  |  |  |  |  |  | .98*** |
| Anxiety | .49*** | .31*** | -.05* | .19*** | .44*** | .78*** |  |  |  |  |  | .98*** |
| Stress | .46*** | .34*** | -.07** | .19*** | .45*** | .85*** | .80*** |  |  |  |  | .98*** |
| ACE | .23*** | .27*** | .00 | .10*** | .23*** | .36*** | .27*** | .34*** |  |  |  | .87*** |
| PCL | .49*** | .34*** | -.05* | .22*** | .48*** | .75*** | .72*** | .75*** | .38*** |  |  | .98*** |
| LEC | .22*** | .28*** | .00 | .14*** | .21*** | .27*** | .24*** | .33*** | .36*** | .31*** |  | .91*** |
| Negative urgency | .31*** | .22*** | -.01 | .05* | .33*** | .39*** | .39*** | .41*** | .23*** | .42*** | .15*** | .97*** |

* p < .05, ** p < .01, *** p < .001
